# Optimization of Soft X-Ray Fresnel Zone Plate Fabrication Through Joint Electron Beam Lithography and Cryo-Etching Techniques

**DOI:** 10.3390/nano14231898

**Published:** 2024-11-26

**Authors:** Maha Labani, Vito Clericò, Enrique Diez, Giancarlo Gatti, Mario Amado, Ana Pérez-Rodríguez

**Affiliations:** 1 Nanotechnology Group, USAL-Nanolab, Departamento de Física Fundamental, Universidad de Salamanca (USAL), E-37008 Salamanca, Spain; idu018684@usal.es (M.L.);; 2 Centro de Láseres Pulsados (CLPU), E-37185 Villamayor, Spain; 3 Instituto Universitario de Física Fundamental y Matemáticas (IUFFyM), Universidad de Salamanca, E-37008 Salamanca, Spain

**Keywords:** electron beam lithography, cryogenic etching, fresnel zone plates

## Abstract

The ability to manufacture complex 3D structures with nanometer-scale resolution, such as Fresnel Zone Plates (FZPs), is crucial to achieve state-of-the-art control in X-ray sources for use in a diverse range of cutting-edge applications. This study demonstrates a novel approach combining Electron Beam Lithography (EBL) and cryoetching to produce silicon-based FZP prototypes as a test bench to assess the strong points and limitations of this fabrication method. Through this method, we obtained FZPs with 100 zones, a diameter of 20 µm, and an outermost zone width of 50 nm, resulting in a high aspect ratio that is suitable for use across a range of photon energies. The process incorporates a chromium mask in the EBL stage, enhancing microstructure precision and mitigating pattern collapse challenges. This minimized issues of under- and over-etching, producing well-defined patterns with a nanometer-scale resolution and low roughness. The refined process thus holds promise for achieving improved optical resolution and efficiency in FZPs, making it viable for the fabrication of high-performance, nanometer-scale devices.

## 1. Introduction

In the rapidly evolving field of nanotechnology, the ability to fabricate structures at the nanometer scale is crucial for advancing a myriad of applications. Among the techniques that have emerged to meet this demand, lithography and cryoetching stand out for their precision and versatility [1]. Lithography, a process that uses electrons to transfer a pattern onto a substrate, and cryoetching, which involves etching at cryogenic temperatures to achieve smooth high-resolution patterns, together provide a powerful toolkit for nanofabrication. This combination is pivotal in the creation of intricate nanometric structures essential for the miniaturization and enhancement of various devices encompassing high-density memory elements, structural elements, and functional surfaces, such as cantilevers or lamellae, as well as the fabrication of arrays of Josephson junctions for quantum computing, to cite just a few examples. X-rays, widely used for their ability to penetrate matter and unveil intricate structural details at electronic, atomic, and molecular levels in space and time, with unprecedented resolution, serve as an indispensable tool across numerous scientific fields, such as materials science, medicine, chemistry, and physics [2,3,4,5,6]. The full exploitation of the latest generation of Free Electron Lasers and synchrotron light sources [7,8,9,10,11,12,13] requires the use of state of the art devices that are able to focus X-rays at their diffraction limit, at a resolution of at least tens of nanometers. In addition to the aforementioned established players in the field, laser-driven X-ray sources are emerging as a promising and rapidly evolving field [14,15,16], which require a set of new tools to take advantage of their capabilities.

A soft X-ray beam can be successfully monochromatized and focused at photon energies below 1400 eV with a high transmittance of up to 20% using reflection zone plates (RZPs), which are made of elliptical zones constructed as laminar grating structures on a mirror surface. Nevertheless, RZPs can only be built for a single photon energy and are extremely chromatic. A reflection zone–plate array (RZPA), which covers a wide bandwidth while maintaining pulse form and flux, is the new solution proposed by Maria B. et al. [17] to mitigate this issue. Unprecedented new methods for examining the electronic structure and dynamics of transition metal systems are made possible by X-ray free-electron lasers, or XFELs. One effective method for these kinds of investigations is L-edge absorption spectroscopy, which has been shown to be feasible at XFELs for both liquids and solids. However, further monochromatization is required, and the necessary X-ray bandwidth is an order of magnitude smaller than that of self-amplified spontaneous emission (SASE). A novel method based on the self-seeding of a linac coherent light source (LCLS) is compared with the L-edge X-ray absorption spectroscopy (XAS) of a prototypical transition metal system, based on monochromatizing the SASE radiation of the LCLS, by Thomas K. et al. [18].

In high-precision optical systems, scanning focused light with corrected aberrations is crucial. Nevertheless, traditional optical systems that depend on extra dynamical correctors to remove scanning aberrations invariably produce unwanted complexity and bulkiness. Xiaotong Li et al. [19] suggest that by rotating just two cascaded transmissive metasurfaces, adaptive aberration corrections can be achieved in tandem with focus scanning. They designed and built two all-silicon terahertz meta-devices that can scan the focal spot with adaptively corrected aberrations. Laser-induced breakdown spectroscopy (LIBS) has been utilized extensively in industrial measurement and monitoring as a vital detecting tool in the age of digital transformation. Combining LIBS technology with other technologies has become an unavoidable trend for present and future development, with the goal of diversifying detection functions. Minchao C et al. [20] thoroughly examine the state of LIBS in conjunction with laser-induced fluorescence spectroscopy, Raman, X-ray fluorescence, acoustic signals, photoacoustic spectroscopy, and hyperspectral imaging, among other methods, and differentiate the respective advantages of LIBS and these techniques.

Grazing incidence mirrors and Fresnel Zone Plates (FZPs) are currently the optical devices that are able to reach the highest imaging resolutions in the soft X-ray range [21,22,23], since FZPs offer an unrivaled performances in terms of compactness, as well as a superior stability with respect to environmental parameters. This ability to establish fine structural details in zone fabrication not only facilitates high-resolution focusing but also eliminates the need for complex multilayer reflective coatings or the stringent alignment requirements inherent to RZPAs. As a result, FZPs provide a more efficient and practical solution for the high-resolution demands of soft X-ray microscopy, achieving a greater imaging performance in this spectral range.

Various fabrication techniques have been employed by researchers to create Fresnel zone plates (FZPs) from a wide range of materials, with diverse dimensions. For example, Di Fabrizio et al. [24] fabricated a silicon nitride and nickel FZP with a diameter of 5000 µm and a thickness of 1.3 µm using electron-beam lithography. Rogers et al. [25] utilized ion-beam milling on aluminum to produce a significantly thinner FZP (0.1 µm) with a diameter of 40 µm. Kong et al. [26] employed photolithography to create an ultra-thin graphene FZP with a diameter of 100 µm and a thickness of less than 0.005 µm. Li et al. [27] achieved a 10 µm thick carbon nanotube-based FZP with a diameter of approximately 650 µm using lithography. Additionally, Zheng et al. [28] and Low et al. [29] employed femtosecond direct laser writing (FsDLW) on graphene oxide, utilizing adaptive optics and a 3D stage or Galvano scanner, to fabricate FZPs with thicknesses of 0.28 µm and 0.9 µm and diameters of approximately 8 µm and 656 µm, respectively.

The key factors and challenges in FZP manufacturing can be condensed during extreme control (i.e., required resolutions at the nm-scale) of the manufacturing process of a full 3D device over a range of tens of µm (i.e., the overall transverse size) and thicknesses at the µm scale. As a starting point in the design of the FZP, a mean photon energy $E_{0}$ and focal length will be defined [30,31,32]. The FZP topology used throughout this work is composed of concentric zones, alternatively illuminated by photons with a half wavelength of phase difference. The focusing effect is achieved by alternating zones absorbing and transmitting the incoming radiation. The absorbing zones will be characterized by a given material of a constant thickness devoted to attenuating radiation. Transmission zones would be made out of a material thin enough to minimize X-ray absorption. Once the material composition of the absorbing zones is chosen, the photon mean energy thickness *t* of the FZP is set. The radius of the concentric *n*th zone is given by the following expression:

(1) $$r_{n}^{2}=n\lambda f+\frac{n^{2}\lambda^{2}}{4}$$

where (*n*) is the zone number, (λ) is the radiation wavelength, and (*f*) is the focal length. By increasing *n*, Equation (1) encompasses a constant area for each zone, while the spacing between adjacent zones $\Delta r_{n}=r_{n}-r_{n-1}$ would progressively shrink. Clearly, the technological limit of the fabrication process would determine the width of the outermost zone Δr, and hence, the diameter of the FZP, its numerical aperture (NA), and the imaging resolution. The Δr limit would also set the aspect ratio of the FZP, t/Δr. This is an important figure of merit, as it summarizes the most challenging aspects of FZP fabrication, which make the outermost zones challenging to manufacture, and extremely fragile.

There have been various approaches to fabricating such FZPs, such as photolithography [33], focused ion beam milling [34,35], interference lithography [36], and electron beam lithography (EBL) [37]. Achieving the nanometer-scale resolution required for cutting-edge applications necessitates the development of diminutive lenses with sharp and smooth edges. To fabricate such high-precision FZPs, it is imperative to push the limits of electron beam lithography (EBL). EBL, known for its exceptional resolution, can define the intricate patterns required for FZPs. When combined with cryoetching, the production of FZPs with sharp and smooth edges would become significantly more feasible. However, achieving defined nano-metric patterns with EBL presents challenges. During exposure, the energy deposition distribution within the resist significantly influences the patterning performance of the masks produced. Incident electrons penetrate the resist and substrate, undergoing scattering and energy loss [38], which can hinder the technique’s applicability at the nanoscale. To mitigate these issues, the implementation of a capping layer is crucial. A capping layer helps minimize the backscattering effects, ensuring that electrons are effectively absorbed or scattered away from the underlying layers, thereby reducing the risk of unwanted exposure and pattern distortion [39,40]. Various materials, such as Al-Cu, Ti, TiN, and Cr, have been evaluated for this purpose, with Cr emerging as one of the most promising options [41,42].

In this study, we thoroughly report on the fabrication steps of a Si-based FZP structure, with 100 differentiated zones, a lens diameter of 20 µm, and an outermost zone width of 50 nm and thickness of 4.3 µm. Our goal is to investigate the limits of the combination of electron beam lithography (EBL) and cryoetching for the design of structures that might be used in prototypical FZPs when conveniently scaled, as the employed aspect ratio of 86 is quite challenging and poses severe issues in the manufacturing process as well as in the handling of the FZP. As an example, by using the scaling law given by Equation (1), we could use the structure synthesized directly as a Si-FZP for photons of 1.2 keV, for a focal length of 970.87 µm, and for 3 keV (with 2.5 times the initial focal length), or, conversely, as a masking pattern used to make the deposition of conventional absorbing materials (e.g., Ni, Au, Ta, W) suitable for harder X-rays (e.g., 9 keV). We developed a robust process utilizing EBL to ensure a fine definition of intricate micrometric structures is achieved. This method is combined with a chromium mask, which guarantees the successful fabrication of a vertical-step FZP comprising a large number of differentiated zones via cryoetching [1]. This approach aims to significantly improve the focusing performance of the FZP. Building on the results presented by K. Parfeniukas and J. Rahomäki [43], who demonstrated an improved method for the nanofabrication of high-aspect-ratio tungsten structures for high-efficiency hard X-ray nanofocusing zone plates, we addressed the issue of the efficiency loss towards the edge of the zone plate. This loss was partly attributed to flaws in the vertical zone profile. In our work, we demonstrate how the use of cryogenic etching for the FZP allows for the precise control of the side wall angle and the roughness at the edges [44], which are crucial for achieving a high lens efficiency

## 2. Fabrication and Discussion

### 2.1. PMMA Mask Preparation

Here, we report a process based on EBL, aiming for the sharp definition of micrometric structures to create an FZP suitable for a wavelength of 1.03 nm (1200 eV), which would allow for an optical resolution of 61 nm and could accordingly be scaled at higher photon energies, as previously stated. The rest of the parameters of the FZPs’ design are detailed in Table A3 in the Appendix C. In order to define the microstructures, resist PMMA layers were deposited on freshly cleaned 550 µm thick silicon substrates, which served as the template for the design of the FZP. The standard cleaning process of the substrate was achieved through rinsing in acetone and isopropanol (IPA), followed by a plasma cleaning treatment for 5 min, utilizing a power of 29.6 W, and an oxygen flow rate of 30 mL/min, while maintaining a stable base chamber pressure of 1000 mTorr. The PMMA layers were spin-coated using a resist solution (2% PMMA in chlorobenzene), followed by a soft-bake at 160 °C for 10 min [1].

**Figure A1:**
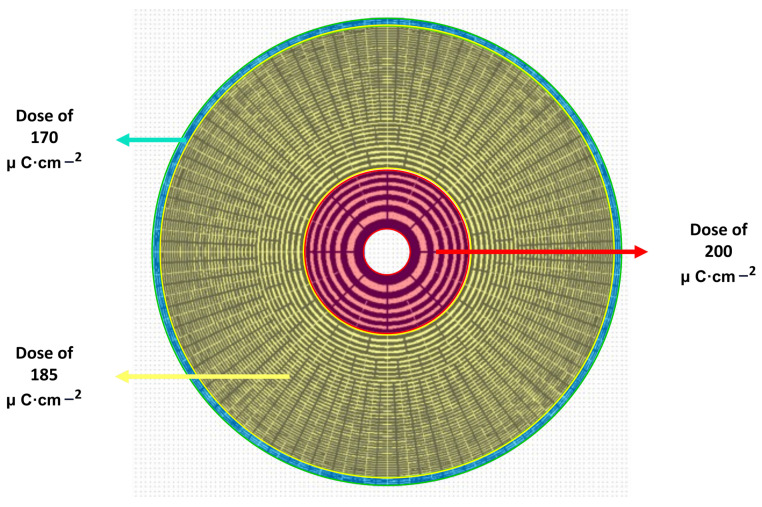
Final computer-aided designs (CAD) where different doses of electron beam exposure are applied to different parts of the pattern: the first six rings receive a dose of 200 μC·cm^−2^, the last three rings receive a dose of 170 μC·cm^−2^ and rings in the middle receive a dose of 185 μC·cm^−2^.

The design of the Fresnel zone plate pattern was constructed in the GDSII file format using the ELPHY PLUS software release 6 SP 6.0 (see Figure A1 in the Appendix A), and considering various parameters, since our goal is to design structures that could be of use in focusing X-rays. For the selection of substrate materials for the FZP with a high X-ray transmittance, low absorption, and an appropriate refractive index, commonly utilized options include diamond and silicon nitride [45,46]; the selection of these substrate materials is fundamental. Spatial resolution is determined by the width of individual zones using the expression δr=λ/2(NA), and strongly depends on the fabrication process and methods. The overall efficiency is dependent on parameters such as the number of zones, their thickness, and their accurate placement. A zone plate’s resolving power depends on the illumination conditions, i.e., the numerical aperture (NA) of the condenser system [47]. Specific zone plate parameters, including the number of zones (N), the zone diameter (D), the zone thickness (t), and the outermost zone width (Δr) require a delicate balance between spatial resolution and efficiency.

Once the design was complete, the subsequent electron beam lithography exposures were carried out with a scanning electron microscope (SEM) Sigma, manufactured by Zeiss (Jena, Germany). The EBL process was executed employing a 15 keV electron beam energy and a 7.5 µm column aperture, maintaining a typical beam current of 0.016114 nA. Specific details of the EBL settings are found in Table 1, ensuring consistency and reproducibility in the manufacturing process.

**Figure 1:**
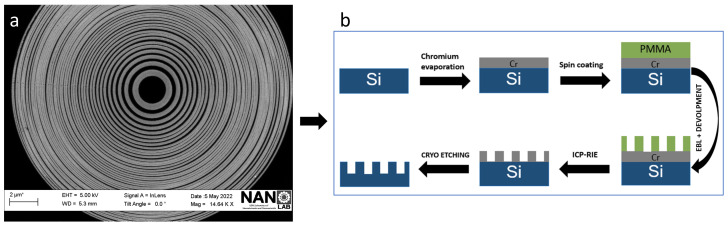
(**a**) Electron micrograph of the original PMMA, designed to be used as a mask for the fabrication of a standard FZP with 100 concentric regions. This initial approach, commonly used in larger FZPs, can lead to the occurrence of structure collapse at the outermost zones. (**b**) Fresnel zone plate manufacturing steps, including all the necessary measures to improve the final structure. This sequence will produce the final structures.

Following the EBL exposure, the sample underwent a sequence of post-processing steps. It commenced with a 45 s immersion in a 1:3 mixture of MIBK (4-Methyl-2-pentanone) and isopropyl alcohol (IPA), designed to develop the patterned resist. Afterwards, the sample was immersed in isopropyl alcohol (IPA) for an additional 45 s, ensuring the removal of any residual materials. The fabricated structures were thoroughly examined using Scanning Electron Microscope (SEM). A typical FZP structure produced by EBL is shown in Figure 1a, where an already developed PMMA mask is presented. The outermost zones’ decrease in width poses a challenge known as the collapse problem in micrometric-size structures due to their high aspect ratios. Figure 1a depicts a concentric FZP mask made of PMMA, showing a large portion of undesired, overexposed (and hence collapsed) areas at the outermost zones due to the undesired over/underexposure. The collapse problem is caused by the proximity effect [48,49,50]. This significantly affects each pattern and its neighbors and leads to pattern interference during exposure. When an electron beam and a substrate come into contact, this leads to the production of forward- (electrons are deflected at tiny angles as they enter the substrate and resist) and backscattering (electrons are usually deflected in a large angle) electrons, which can result in the resist material being exposed beyond the initial point of impact. To be more specific, during the EBL manufacturing process, the electron gun uses a series of lenses and apertures to directly imprint patterns on wafers. When a primary electron beam shoots from the electron cannon and hits the resist and substrate, the electrons may scatter, and the scattered electrons produce backscattered electrons. Rescattered electrons can be created when these backscattered electrons strike the objective lens’s bottom [51]. As a result, the resist is inaccurately exposed in areas that are close to those that the electron beam is targeting due to the electrons that are dispersed within the resist and from the substrate. This collapse of the PMMA structures after their development, as well as the lack of definition of the walls, led to an improvement in the optimization process of this work. The final procedure is sketched in Figure 1b.

**Figure A2:**
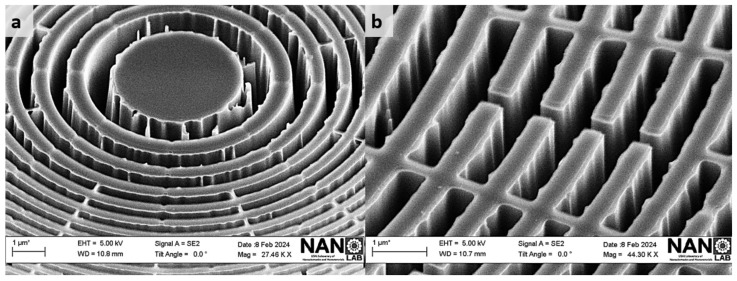
Tilted (60°) scanning electron microscope (SEM) images were used to observe the center (**a**) and outer patterns (**b**) of the Fresnel lens. The etching process involved using the resist PMMA as a mask and the following etching conditions: pressure of 10 mTorr, RF/ICP power of 4/1000 W, a gas mixture of SF_6_/O_2_ with a flow rate of 60/15 sccm, and a temperature of −120 °C. The etching test was carried out for 2 min.

To address these initial collapse problems more effectively, we combined two strategies. First, a thin chromium layer (typically of 7 nm) was deposited directly on top of the bare Si, which avoids the majority of undesired electron backscattering [52,53,54]. Directly using the PMMA layer as a mask, without an additional chromium layer for silicon etching, affects the resulting patterns and leads to roughness and undulation in the side walls (as shown in Figure A2 in the Appendix B). Secondly, novel structures containing element fragmentation and reinforcing structures were designed and implemented, alongside different doses for the lithography. These were applied to different regions of the pattern, allowing for better control and the optimization of scan directions [55]. Chromium is widely used as a hard mask material for plasma etching for a number of reasons; it is known for its ease of deposition through physical vapor deposition methods, the etching can easily be removed, and it has a low atomic mass, making it easier to image pre-patterned metal structures for alignment purposes. Moreover, it is also an effective hard masking material for dry etching, as it exhibits high sputter resistance and selectivity in fluorine and bromine chemistries [56,57]. These advantages allow for chromium to serve as an etching mask for various materials, including silicon, SiO_2_, ZnO, Si_3_N_4_, TiO_2_, MoSi, and GaAs [58].

**Figure 2:**
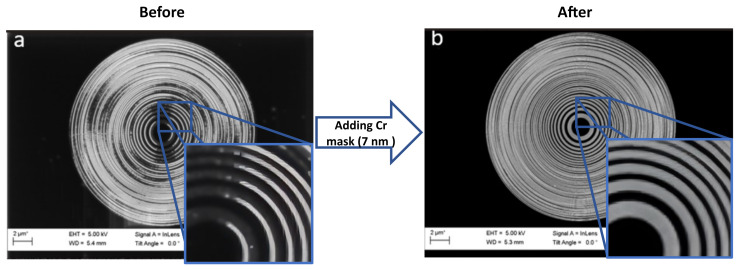
Scanning electron microscope (SEM) images of FZP using a concentric design (**a**) without and (**b**) with an extra Cr-layer (7 nm) on top of the Si for a dose of 160 μC·cm^−2^. Remarkable improvements in the final FZP can be seen upon the addition of the chromium layer to the silicon chips, which effectively addressed several issues, resulting in a significant enhancement of the overall pattern quality. However, some collapsing can still be observed, particularly in the outermost regions where the separation between concentric rings became too narrow.

The effect of the Cr layer is immediately observed. Figure 2a displays a concentric PMMA mask for the generation of FZP without the previously deposited Cr on top of the bare Si, showing a large portion of undesired, overexposed (and hence collapsed) areas throughout the whole structure where the pattern was harmed by the excessive clearance of PMMA. The observed overexposure is directly linked to the electron dose that is applied; in this case, a uniform dose of 100 μC·cm^−2^ was administered across the entire mask, from the center to the edges. As shown in the zoomed-in section of Figure 2a, the central region exhibits signs of overexposure. On the other hand, Figure 2b presents the final patterned resist mask using the same initial CAD design and the same dose of 100 μC·cm^−2^, with a previous Cr layer showcasing a much-improved final structure without any distinguishable damaged areas. The improvement was most evident in the zoomed-in area of Figure 2b, where collapse is absent and the overexposure has been eliminated. The metal coating serves two purposes, i.e., it eliminates the charge buildup that occurs during EBL and allows for a more precise beam-focusing without obstructing the patterning process and, secondly, it facilitates the better adherence of the PMMA [59,60].

**Figure 3:**
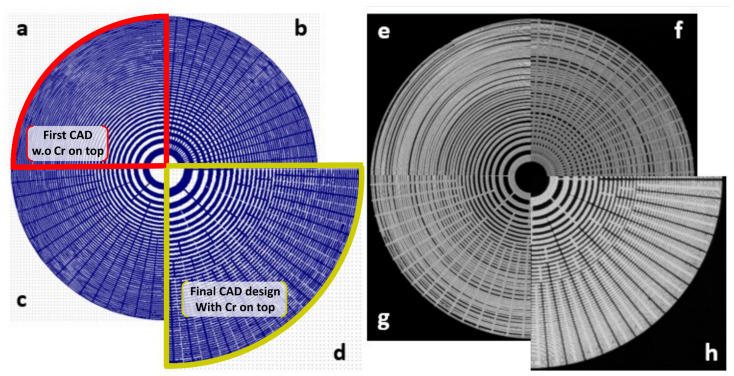
(**a**) Computer-aided common design (CAD) of a Fresnel Zone plate with 100 concentric zones. (**b**) modified CAD design with the inclusion of 72 segments, each 0.05 µm wide, with a five-degree angle spacing from the first to the last zone. (**c**) The new segments were modified in the CAD design such that eight of them began in the first zone and ended in the tenth zone, while the remaining segments began in the sixth zone and ended in the last zone, with varying lengths. (**d**) A CAD design that is akin to the one presented in (**c**) but with a set of intermediate segments to the lower local exposure observed there. (**e**–**g**) are scanning electron microscope (SEM) images of the already developed PMMA using designs (**a**–**c**), respectively, which still shows the presence of resist collapse even after adding extra segments both radially and circularly. Panel (**h**) displays the final PMMA mask on top of Cr/Si, with no traces of proximity effect within their outermost regions. This final mask was retrieved from the CAD presented in panel (**d**) and demonstrates the effectiveness of this final morphology, where the improved design generates the final well-defined patterns.

The four different CAD geometries developed in this work are displayed in Figure 3a–d, with their already developed PMMA counterparts shown in panels Figure 3e–h, respectively. Firstly, from the common stem of the concentric-like shape, we added 72 radial lines with a spacing of five degrees, which helped to ease the majority of the overexposure at the outermost regions, as shown in Figure 3b. This was translated onto a final PMMA mask in Figure 3f. Figure 3c shows an evolution of the design shown in Figure 3c, where not all 72 lines start from the first radial structure but commence at different distances from the origin, alleviating the undesired overstructure that occurs close to the center. This design still provokes collapse and causes stress to the final PMMA mask next to the edges, as shown in Figure 3g. Finally, Figure 3d displays the final CAD design where the majority of the external concentric rings are interrupted in the vicinity of the radius. Different doses of electron beam exposure were applied to different parts of the pattern, allowing for better control and the optimization of scan directions [55]. Three different dosages were employed in the four CAD designs (Figure 3): 200 μC·cm^−2^, 170 μC·cm^−2^, and 185 μC·cm^−2^. These dosages were used for the middle six rings, the last three rings, and the rings between these groups, respectively. This technique helps mitigate the collapse problem by minimizing the interaction between neighboring patterns and reducing the overall stress on the resist material.

**Figure 4:**
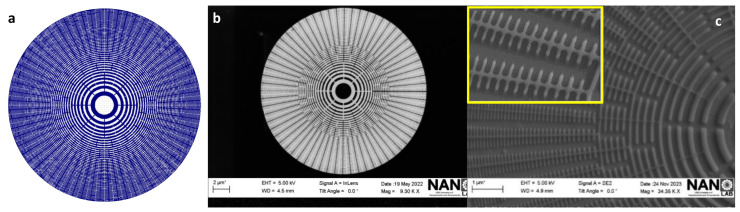
(**a**) Final computer-aided designs (CAD). (**b**,**c**) SEM images that display the resulting PMMA mask from afar and a zoomed angle of view, highlighting the removal of collapse.

The final optimized PMMA mask for Cr/Si, presented in Figure 3h, shows an overall improvement oin the desired structure, with no traces of overexposure or collapse. These results demonstrate that the strategy of combining the metal coating and element fragmentation was successful in reducing backscattering during EBL exposure. This approach succeeds in minimizing the proximity effects and leads to an efficient optimization of the PMMA mask. A more detailed picture of the produced mask is shown in Figure 4. The optimized CAD design is shown in Figure 4a while an SEM image of the final mask is displayed in Figure 4b, from which a zoomed area, Figure 4c, displays intact and well-defined structures, where no over-etch effects are found.

**Figure 5:**
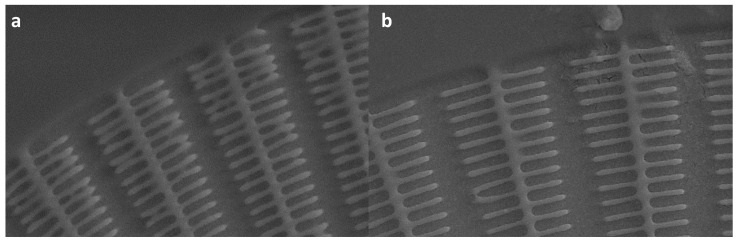
Scanning electron microscope (SEM) overviews of comparative analysis for the outer patterns without (**a**) and after (**b**) the DESCUM etching process using a pressure of 60 mTorr and an oxygen flow set at 50 sccm: Emergence of Clearly Defined Patterns.

A detailed examination using the SEM of the produced PMMA structures is fundamental to enhancing the outer zone patterns in order to identify significant pattern collapse, as was observed in the images shown in Figure 5a. This phenomenon was produced due to the presence of residual resist material during the development stage. The constraints imposed by the narrowest separation between patterns hindered the developer’s efficacy. To overcome this challenge and achieve optimization, we employed DESCUM etching techniques. This strategic approach not only facilitated the removal of the persistent resist material but also played a pivotal role in refining the overall pattern morphology.

The DESCUM process serves as a critical step in the integrated circuit manufacturing process, specifically designed to eliminate residual organic residues that persist after the photolithography stages. It consists of a low-temperature, oxygen-based plasma process operating at low power, and is specifically engineered to remove a few hundred targeted angstroms of resist material. This process guarantees a clean and uncontaminated surface, setting the stage for the subsequent critical process steps, including wet and dry etching. By effectively eradicating organic residues, DESCUM improves the overall quality of the structures [61].

The DESCUM process was carried out in the PlasmaPro 100 Estrelas system, (Oxford Instruments Plasma Technology, North End, Yatton, UK) where the specific etching parameters employed in this study included a pressure of 60 mTorr, an RF power of 50 W, O_2_ flow set at 50 sccm, and a temperature maintained at 25 °C. The etching duration was optimized at 5 s. DESCUM using oxygen plasma produces radical oxygen species to chemically eliminate the silicon wafer’s resist layer, where oxygen molecules can be broken down by intense electrons in the plasma to produce reactive oxygen atoms; moreover, oxygen plasma ashing produces non-toxic byproducts. In comparison to the wet etching method, it is more ecologically friendly. The findings are displayed in Figure 5b, which displays the precisely defined and narrow patterns of the outermost zone, contrasted with the scenario in which DESSCUM was not used, indicating that the decision to use DESCUM was successful in eliminating the remaining resist, which was challenging to eliminate when using only development. This result shows that the PMMA 2% resist mask is effective and ready for the next step of chromium etching. The strong and controlled DESCUM process sets the stage for a smooth fabrication, guaranteeing the creation of high-quality, well-defined patterns.

### 2.2. Chromium Mask Fabrication

Once the PMMA mask has been produced, ensuring the high quality of the desired structures, the Cr layer is ready to be etched, and the pattern is ready to be transferred in order to create the final mask (as shown in the schematic in Figure 1b)

**Figure 6:**
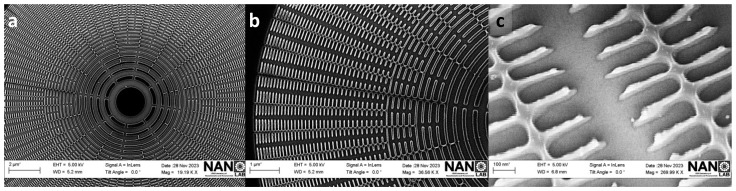
A comprehensive view from above reveals the central zone (**a**), outermost zone (**b**), and pattern details of the FZP (**c**) after 55 s of room-temperature dry etching of chromium at a chamber pressure of 12 mTorr. The etching process employed a gas flow combination of 2 sccm of O_2_ and 14.7 sccm of Cl_2_. In panel (**a**), the innermost region of the FZP is clearly visible, showcasing the high-quality definition of the desired structures, along with a Moiré pattern that contrasts with the highly defined symmetrical structures. Panel (**b**) presents the outermost region of the structure, where the nm-scale patterns remain well-preserved after the Cr-etching. Finally, panel (**c**) provides a detailed view of the outermost region of the Cr mask, where the 7 nm thick metal remains intact as a hard mask, with a thin layer of residual PMMA.

To transfer the patterned features of the thin PMMA layer to the underlying material with a Cr mask, we used the Inductively Coupled Plasma Reactive Ion Etching (ICP-RIE) technique [62]. The first chromium etchings were developed at room temperature. To achieve optimal results, a specific gas flow rate comprising 14.7 sccm of Cl_2_ and 2 sccm of O_2_ was maintained within the chamber. Additionally, the chamber pressure was regulated at 12 mTorr, the ICP power set at 600 W, and the RF power at 8 W. (all other Cr etching recipes tested in this study are depicted in the Appendix B, Table A1). The duration of the processes varied between 30 and 55 s, ensuring the desired pattern was accurately transferred to the underlayer material. Following the completion of the chromium etching, a thorough cleansing was achieved, utilizing 100 sccm of Argon (Ar) for one minute. This step ensured the removal of any remaining trace materials, resulting in clean and overall well-defined patterns, as depicted in Figure 6, which clearly shows the process of successfully etching away the chromium layer. The zoomed area in (c) depicts some fine pattern details where the structures are well-defined and suited for their use as FZPs. By using the method presented above, the etching rate for chromium was 7.64 nm/min. We were able to completely etch the 7 nm chromium film after 55 s. Regarding the PMMA (with a thickness of 83 nm), the etching rate was 89 nm/min. This indicates that there was a very small amount of PMMA left (about 2 nm) after a 81 nm etch over 55 s, as seen in Figure 6c. An ultra-thin layer of PMMA (around 2 nm thick) remains on top of the Cr mask.

### 2.3. Silicon Cryogenic Etching

In order to overcome the faulty definition of the outermost zones of the FZP, where nm-size structures are required, we extensively made use of cryo-etching, allowing for the production of clean structures with highly controlled edge shaping. In silicon-based devices, this has been successfully implemented with defined low-roughness side walls [63], generating clean samples with a high aspect ratio [64] in nanometric structures [44].

The cryo-etching was performed using an Inductively Coupled Plasma (ICP) Reactive Ion Etching (RIE) system; specifically, the Oxford Instruments Plasma Pro 100 Cobra (Oxford Instruments Plasma Technology North End, Yatton, UK). This process involved the controlled introduction of sulfur hexafluoride (SF_6_) and oxygen (O_2_) gases and was executed at extremely low cryogenic temperatures, with the operational temperature maintained below −100 °C [65]. For a comprehensive overview of the typical operational conditions employed in our cryo-etching process, please refer to Table 2. All Si etching recipes tested in this study are shown in the Appendix B, Table A2

**Figure 7:**
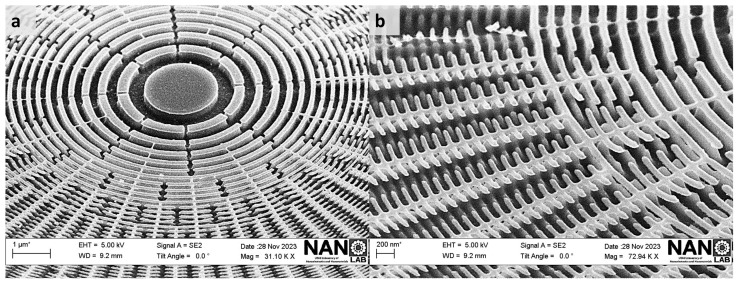
Inclined view (60°) of the FZP after 15s of the cryo-etching of the silicon layer under the chamber pressure of 10 mTorr and −110 °C with the SF_6_/O_2_ gas flow rate of 60/8 sccm, ICP power of 1000 W and RF power of 4 W.T. (**a**) SEM image depicts silicon etching results highlighting noticeable undercutting. (**b**) displays bending instances in our chromium mask.

**Figure A3:**
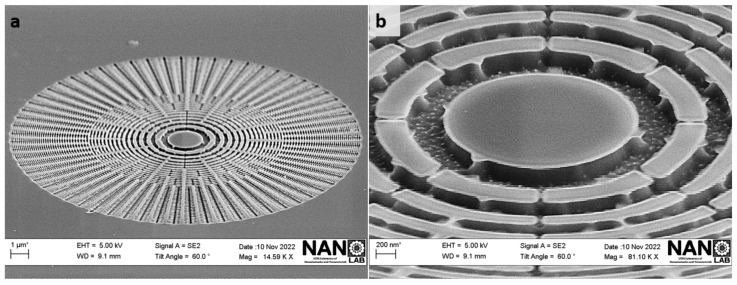
Tilted (60°) Scanning electron microscope (SEM) images captured from a distance (**a**) and close-up (**b**), showing FZP with 100 zones, a diameter of 20 µm, and an outermost zone width of 50 nm after 15 s of silicon etching with an increase in oxygen gas flow to 12 sccm and reduction in the etching cryo-temperature to −120 °C. Under-etching (negative taper) is slightly visible.

**Figure A4:**
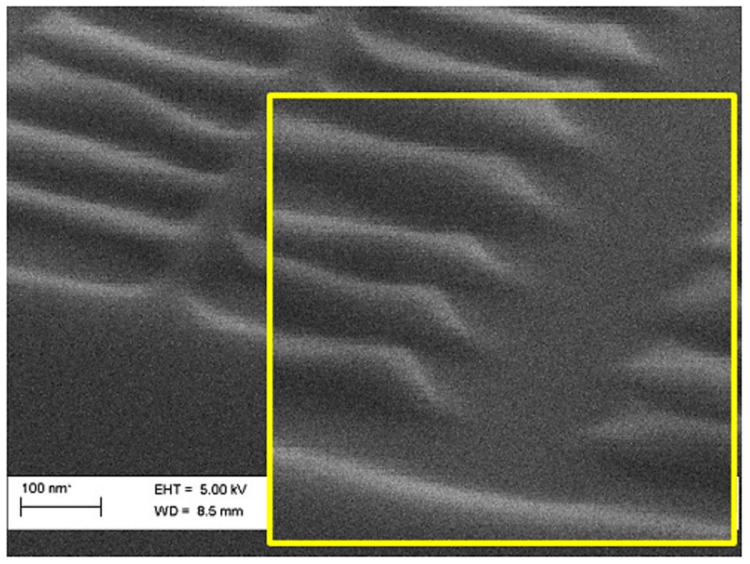
A titled (45°) Scanning Electron Microscope (SEM) image captures the etching profile of the outer zone after a 15 s etching process employing 18 sccm of oxygen flow and a cryogenic temperature of −120 °C. In the zoomed framed image with yellow, the resulting patterns exhibit an over-etched (positive taper) characteristic, showcasing a broader top compared to the bottom. This observation aligns with the effects of the increased oxygen flow and the extreme cryogenic temperature, which collectively contribute to the pronounced widening of the etched structures.

**Figure A5:**
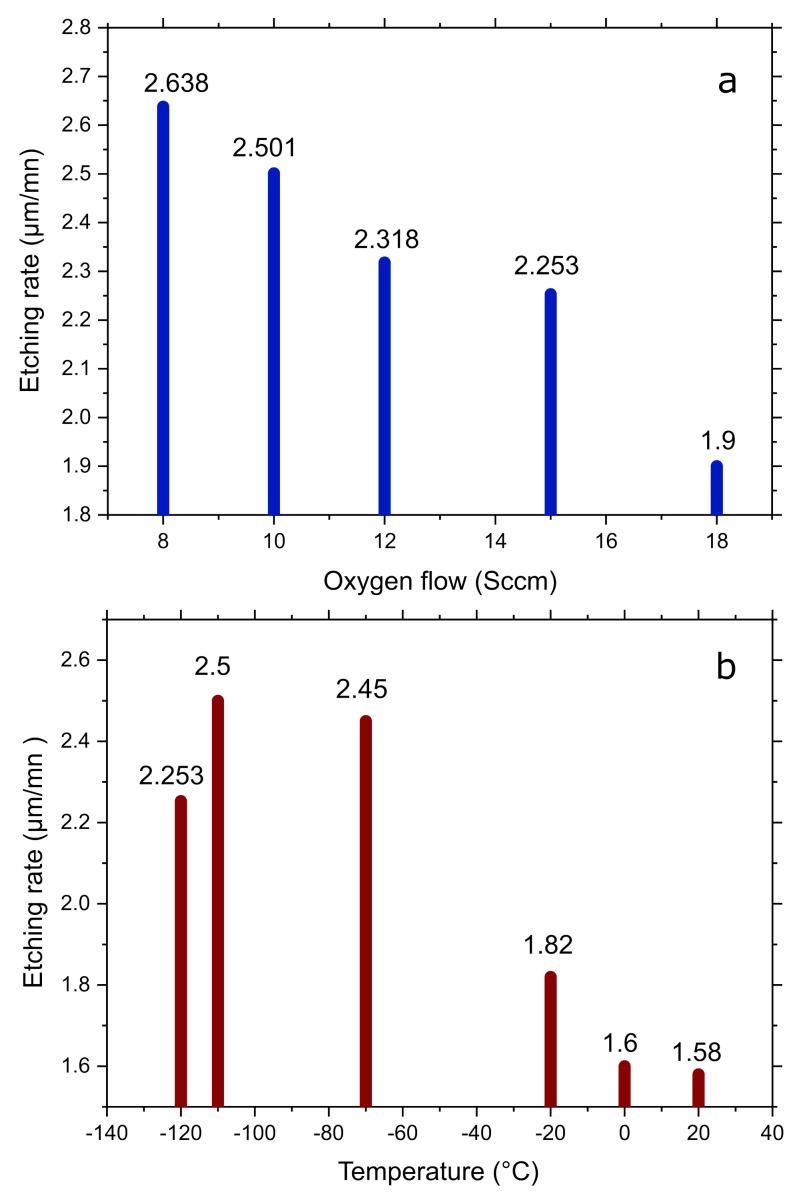
Result analysis of the silicon etch rate in the cryogenic etching process in relation to (**a**) $O_{2}$ gas flow at a constant temperature of −120 °C and (**b**) temperature with constant $O_{2}$ flow of 15 Sccm, where all the etching parameters were maintained, and only the temperature and the oxygen flow were changed.

**Figure A6:**
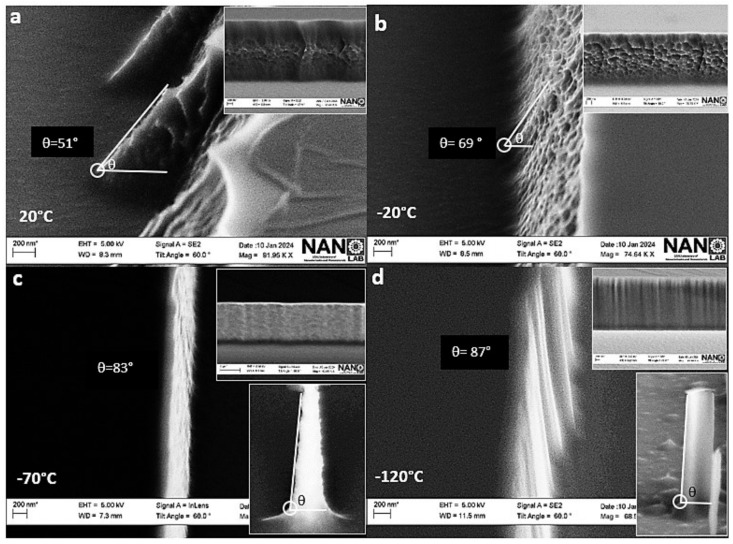
Scanning electron microscope (SEM) images provide a detailed analysis of the etching profile at various temperatures. The images capture the side walls from different angles, showcasing the etching process in a comprehensive manner. At room temperature (20 °C) and at −20 °C, respectively (**a**,**b**), the SEM images reveal that the side walls appear extremely rough. Over-etching is clearly visible, indicating an intense etching process. The large illustration in the SEM image displays the side walls from a side angle, providing a wider perspective of the etching profile. In contrast, at low temperatures (−70 °C and −120 °C) respectively as seen in (**c**,**d**), the side walls undergo a significant change. The SEM images show the softer texture of the sid ewalls, indicating a less aggressive etching process. The small illustration in the upper right corner of the SEM image showcases the side walls from the front angle, offering a closer look at the etching profile. Furthermore, at these low temperatures, the etching tends to be more vertical in nature. This vertical etching pattern is clearly depicted in the small illustrations in the right bottom corner of the SEM image. The side walls appear smoother and more uniform, suggesting a controlled and precise etching process.

Under-etching, the lateral erosion of material beneath a mask during etching, can occur in cryogenic silicon etching. This effect, resulting from lateral etching under the mask, leads to side wall tapering or undercut areas. During cryogenic reactive ion etching (RIE) at low temperatures, the isotropic etching process causes noticeable lateral and undercutting, especially in narrow designs like our lens, with an outer zone width of 50 nm. Strong under-etching can be seen in the structures depicted in Figure 7. This under-etching made the patterns thinner and brittle, which damaged the narrowest lines and led to the bending of the Cr mask. By adjusting the oxygen flow or temperature, we can manipulate the thickness and hardness of the passivation layer (SiO_x_F_y_). This, in turn, affects the resulting etch profiles and etch rate (see Figure A5 and Figure A6 in the Appendix B). Increasing the oxygen flow or reducing the temperature leads to thicker and harder passivation layers, resulting in positive etch profiles (as displayed in Figure A4 in the Appendix B). Conversely, reducing the oxygen flow or increasing the temperature results in thinner and softer passivation layers, leading to negative etch profiles [66] (again, refer to Appendix B, Figure A3). To this end, we performed a thorough study of the etching rates as a function of temperature and gas flow, as introduced in the Appendix B.

Lowering the wafer temperature to cryogenic levels reduces the volatility of the SF_4_ reaction product and enhances the passivisation process [67]. Consequently, etching occurs only in the direction of ion bombardment, resulting in a higher level of anisotropy as the base sustains higher kinetic energy [68]. In this area, the passivation layer is removed, exposing the silicon, and etching proceeds accordingly. We determined that by reducing the etching cryo temperature to −120 °C and increasing the oxygen gas flow to 15 sccm, while maintaining the other parameters as previously specified (see Table 3), we can achieve deeper and steeper trenches, as required for the generation of FZPs.

**Figure 8:**
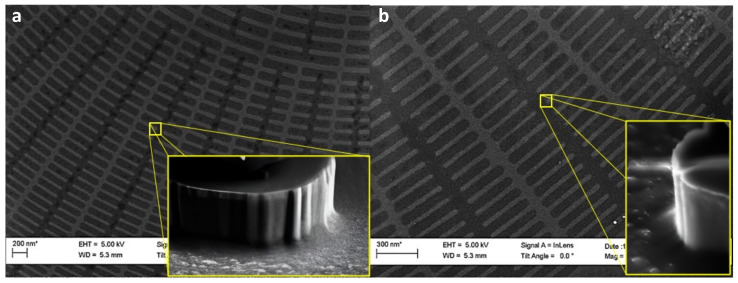
Scanning Electron Microscope (SEM) images (**a**) from the left side and (**b**) from the right side of the lens showcase the etching profile of patterns after a two-minute etching process, utilizing a 15 sccm oxygen flow and a cryogenic temperature of −120 °C. The insets in both images clearly illustrate the vertically etched features and precisely defined patterns resulting from the specified etching conditions.

The resulting vertically well-defined and intact structures are shown in Figure 8, which presents SEM images of the etching profile after a two-minute process, clearly illustrating the well-defined patterns. A standard cleaning process of rinsing in acetone and isopropanol would complete the process by removing the remaining PMMA layer. Keskinbora et al. reported the fabrication of FZPs suited for soft X-ray microscopy with a similar wavelength (1.2 keV), which were able to achieve a spatial resolution in the first order of 61 nm [69]. In those works, trapezoidal-like profiles were obtained via the etching process, which were detrimental for the fluence found in the first order due to the lower lens efficiency provoked by the nonvertical feature profiles and the roughness at the edges [23]. This reduction in the performance of the FZP provokes an increment in the measurement dwell time, which can be overcome using the sharper structures that we fabricated. Moreover, with the correct definition for the outermost vertically well-defined structures obtained via cryo-etching, we are able to increase the lens efficiency [23], overcoming other dry etching methods [70] where a thorough etching was not achieved for the comparable outermost zone widths, which is indispensable for the fabrication of an efficient FZP [71].

**Figure 9:**
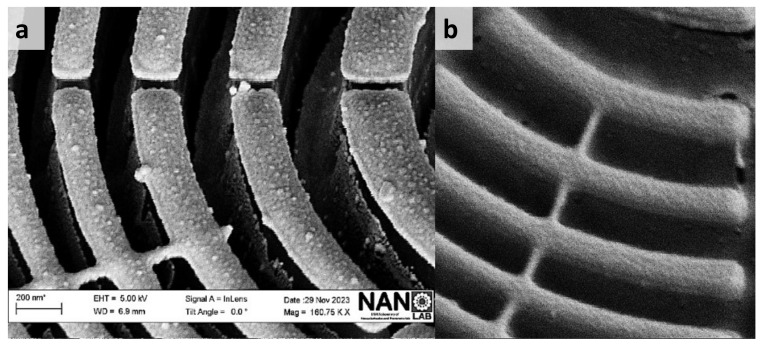
The scanning electron microscopy images provide evidence of the outcome of the shadow mask technique. Image (**a**) illustrates the FZP patterns after the evaporation of a 20 nm layer of gold that covers them. Image (**b**) shows the resultant silicon patterns after the cryogenic etching of the silicon for 3 min. Here, it is apparent that the patterns have become deeper without under-etching.

Finally, we describe the shadow mask evaporation process, an additional technique used in our study to enhance the etching results. Specifically, we applied the shadow mask method during Fresnel lens fabrication to increase pattern depth. After two minutes of silicon etching, we deposited a gold layer over the lens to protect the existing patterns, allowing for further etching to achieve a greater pattern depth. During the evaporation process, the sample was tilted approximately 30 degrees to target and coat the side walls that were not protected by the chromium mask, thereby enabling further silicon etching. In our e-beam evaporator, the base pressure of the main chamber is normally in the range of $10^{-10}$ mbar. The sample is loaded from the load chamber when the pressure reaches a value of $10^{-6}$ mbar. Using our Telemark e-beam gun, we applied an acceleration voltage of 8 keV to direct electrons onto the crucible. The gold, pre-melted for optimal evaporation, required a gradual increase in the emission current to prevent material spitting. In our setup, gold begins to evaporate at an emission current of 160–170 mA; however, reaching 220 mA is necessary to achieve a stable deposition rate of 0.1 nm/s. Upon completing the final deposition, we transferred the sample from the main chamber to the load lock and powered down the system. Applying a 20 nm thick gold layer to seal the etched silicon patterns resulted in a significant improvement in the etching process. This enhancement allowed us to create more complex and deeper patterns by extending the etching time beyond 3 min, completely preventing under-etching. As shown in Figure 9, the addition of the gold layer substantially improved the process by protecting the side walls from over-etching. This layer preserved the integrity and shape of the etched patterns, enabling a controlled and precise etching that produced patterns with greater a depth and definition.

## 3. Conclusions

Our research presents an advanced method for crafting nm-size Fresnel zone plates, combining a precise CAD design with etching at cryogenic temperatures, a technique that surpasses traditional etching methods in terms of the definition of the vertical edges of such structures. We demonstrate that this approach is ideal for producing complex structures with high accuracy. We successfully created a silicon FZP with 100 zones measuring 20 µm in diameter, with an outermost zone of just 50 nm wide and a very high aspect ratio, suitable for direct employment at lower photon energies, as well as a deposition mask for high-Z materials at higher photon energies. Our method combines Electron Beam Lithography for a precise microstructure definition with a chromium mask, ensuring flawless FZP fabrication via cryoetching. Through our refinement of the chromium mask and exploration of different designs, we successfully addressed the issue of pattern collapse. Despite the challenges presented by PMMA resist, we effectively utilized this as a mask for chromium layer etching. By employing cryogenic etching techniques and selecting an optimal processing method, we achieved a silicon etch depth of approximately 4.3 µm, along with well-defined vertical side walls. Additionally, the incorporation of a 20 nm thick gold layer, although complex and time-consuming, provided crucial protection to the outermost regions, enabling the production of high-quality patterns with high aspect ratios. This approach effectively prevents undesired under- or over-etching, making it well-suited for generating nm-scale patterns with low roughness profiles.

## Figures and Tables

**Table 1:** Exposure parameters for the e-beam lithography.

E-Beam Lithography Parameters	
Substrate	Si chip 1 cm × 1 cm
Resist	PMMA 2%
Resist thickness	87 nm
Electron beam current	0.016114 nA
Electron beam energy	15 keV
Step size	2 nm
Dwelltime	0.000284 ms
Column aperture	7.5 µm
Z stage position	17 mm
Working distance	5 mm
Exposure field	100 µm × 100 µm
Magnification	1500

**Table 2:** Typical control values for the silicon reactive ion etching, generating underetched profiles, as shown in Figure 7.

Silicon Reactive Ion Etching	
Pressure	10 mTorr
Rf power	4 W
ICP power	1000 W
SF_6_ flow	60 sccm
O_2_ flow	8 sccm
Temperature	−110 °C
Time	15 s
etching rate	2.63 µm/nm

**Table 3:** Selected parameters for silicon reactive ion etching, resulting in vertically etched features and precisely defined patterns, as shown in Figure 8.

Silicon Reactive Ion Etching	
Pressure	10 mTorr
Rf power	4 W
ICP power	1000 W
SF_6_ flow	60 sccm
O_2_ flow	15 sccm
Temperature	−120 °C
Time	2 mn
etching rate	2.25 µm/nm

## Data Availability

Data are available upon reasonable request to the corresponding author.

## Appendix A. Final Computer-Aided Designs (CAD)

The design of the Fresnel zone plate pattern, as depicted in Figure A1, was carefully constructed in the GDSII file format using the ELPHY PLUS software. Practical proximity effect correction involves two main methods: shape correction and dose-scaling. Shape correction entails adding additional structures in underexposed areas to compensate for the distortion caused by proximity effects, while dose-scaling involves adjusting the exposure dose in different parts of the structure. A dedicated software was used to analyze the proximity effects and make appropriate adjustments to the dose [72,73]. To mitigate proximity effects in the design of the Fresnel zone plate pattern, additional intermediate portions were added to the original design. Three different dosages were employed: 200 μC·cm^−2^, 170 μC·cm^−2^, and 185 μC·cm^−2^. These dosages were used for the middle six rings, the last three rings, and the rings in between, respectively.

## Appendix B. Etching

### Appendix B.1. Etching Recipes

The process of etching begins with the pattern being transferred from the resist layer to the chromium layer underneath, which serves as a mask for the silicon–reactive ion etching (RIE) procedure. During the reactive ion etching (RIE) process, it is crucial to find the ideal method that allows for the chromium mask to remain intact until the completion of the RIE without adversely affecting the resist or the thinning of the chromium layer. It is important to ensure that the resist layer is completely removed in the areas where etching is required while simultaneously protecting the underlying chromium layer. Table A1 outlines the specifications used in the different methods that were tested. Each method considered the etching characteristics of the resist and the chromium layer. In accordance with standard laboratory protocol, the sample was etched for a brief duration of 35 s before being examined using scanning electron microscopy. If required, the etching process was extended to achieve the desired outcome. However, during the initial 12 s of etching using the first method, it was observed that the PMMA2% layer was etched before the chromium layer. Furthermore, the second and third etching methods for the Cr layer were found to be ineffective, as they took a prolonged time of 2 min and 1 min 10 s, respectively, leading to the unintended disappearance of our PMMA layer. Ultimately, the fourth method was close to being suitable, but after 57 s of etching, roughness started to occur in the center rings, and there was some burning of the resist layer [74].

Silicone etching with a fluorine-containing gas, like CF_4_, SF_6_, and C_4_F_8_, has been around for decades and is a well-known process used to anisotropically etch silicon [75]. The desired outcomes in silicon cryogenic etching can be achieved by calibrating various parameters, such as temperature, ICP/RIE powers, and chamber pressure, while using a combination of SF_6_ and O_2_ gases [76,77,78]. It is important to carefully control the gas flow to avoid undesired results. In this study, different recipes were tested and are summarized in Table A2. When using oxygen flow rates of 10 and 12 sccm, under-etching was observed. On the other hand, an over-etching effect was observed when using 18 sccm of oxygen.

**Table A1:** Parameters for chromium dry etching recipes that were tested in this study.

Parameter	Recipe 1	Recipe 2	Recipe 3	Recipe 4
Pressure (mTorr)	5	100	12	6.5
Rf power (W)	30	50	10	8
ICP power (W)	500	0	1200	600
Cl2 flow (sccm)	20	17.2	45	44
O_2_ flow (sccm)	20	4	5	6
Temperature (°C)	10	19	50	15

**Table A2:** Parameters for silicone cryogenic etching recipes tested in this study.

Parameter	Recipe 1	Recipe 2	Recipe 3
Pressure (mTorr)	10	10	10
Rf power (W)	4	4	4
ICP power (W)	1000	1000	1000
SF_6_ flow (sccm)	60	60	60
O_2_ flow (sccm)	10	12	18
Temperature (°C)	−110	−120	−120

### Appendix B.2. Silicon Etching Without Chromium Mask

In the cryogenic etching of silicon, the use of plasma etching along with PMMA is not the most popular approach. This is because PMMA has high-resolution capabilities but poor etch resistance [79]. Additionally, it has poor adhesion on silicon [80], making it only suitable as a soft mask in plasma dry etching. In contrast, a hard mask is typically required for prolonged etching. On the other hand, chromium (Cr) is a popular material in modern nanodevice fabrication. It offers several advantages, such as being opaque (useful in optical lithography), conductive (enabling the use of sensors and actuators), and wear-resistant in harsh environments (making it suitable as a hard mask in plasma etching). Chromium also serves as an important adhesion layer for noble metals, preventing dewetting, particularly with gold [81]. Chromium, along with aluminum (Al), is one of the most popular hard mask materials due to its ease of deposition through evaporation or sputtering and its compatibility with chlorine plasma etching for mask pattern fabrication [82]. Figure A2 represents the results of using a PMMA resist as a mask to etch silicon. It is clear that the patterns lack smoothness and the side walls are peeled and rough.

### Appendix B.3. Under-Etching

Under-etching, a phenomenon characterized by lateral material removal beneath a masking layer during the etching process, is a concern in cryogenic silicon etching. In this context, under-etching arises from the lateral erosion of silicon material beneath the applied mask, resulting in the formation of undercut regions or side wall tapering. In cryogenic reactive ion etching (RIE), under-etching is notable during silicon etching at low temperatures. The isotropic nature of the etching procedure induces lateral and undercutting effects, which are particularly pronounced in small patterns with narrow dimensions, as observed in our lens with an outer zone width of 50 nm. The angle of the side walls in cryogenic etched trenches varies depending on the width of the trenches. The leading explanation for this variation is the limited oxygen transport in smaller trenches, necessitating a higher oxygen flow to create the SiO_x_F_y_ passivation layer for trenches with narrower openings. When using a constant ${\mathrm{SF}}_{6}$ to $O_{2}$ ratio that results in vertical side walls for a specific trench size, an increase in trench size will cause the profile to become more upright as more oxygen becomes available for passivation. Conversely, as the trench size decreases, the profile will show undercutting or bowing as insufficient oxygen is reaching the side walls to form the passivation layer [83]. Temperature and $O_{2}$ flow are two obvious characteristics that regulate the trench profile based on the SiO_x_F_y_ passivation mechanism. Undercut has a considerable temperature dependency. The sticking coefficient of oxygen on silicon increases when the wafer temperature drops from +20 to −130 °C, strengthening the passivation mechanism and reducing lateral etching, as occurs in undercut [84].

By introducing 12 sccm (standard cubic centimeters per minute) of oxygen flow and reducing the temperature to −120 °C, a marginal improvement in the under-cutting of silicon was observed. However, despite these adjustments, some bending and under-cutting still persisted, as shown in Figure A3.

### Appendix B.4. Over-Etching

After the elimination of under-cutting in silicone, the most critical factor is over-etching when using 18 sccm of oxygen, commonly referred to as a positive taper. The SEM image in Figure A4 shows a significant positive taper and a narrowing at the lowermost part. A positive taper, in the context of cryogenic silicon etching, refers to a gradual widening of the etched structure towards the top, resulting in a broader top compared to the bottom. This can be observed in the side wall profile of the etched feature and manifests as a progressive increase in the width or diameter of the etched feature as it extends deeper into the silicon material [83,85].

### Appendix B.5. Etching Rate

Figure A5 represents the dependence of the etching rate on oxygen flow and temperature. In Figure A5a, the etching rate decreases from 2.6 to 1.9 µm/mn while the oxygen flow increases from 08 to 18 sccm. A number of chemical reactions occur at the surface of the material being etched, causing the complex phenomenon known as the drop in etch rate. The development of the SiO_x_F_y_ inhibitory coating is thought to be a key factor in this process. The formation of this film is thought to occur when the O_2_ ratio rises to the point where fluorine is replaced, causing a drop in the reactive species. By acting as a barrier, this inhibitory coating lowers the etch rate by stopping the creation of etch products [86,87].

Moreover, if sulfur is present on the surface at low $O_{2}$ concentrations, this can impede the adsorption of oxygen atoms. This will inhibit how fast the etching process occurs. The complexity of etching and the need for an in-depth understanding of its underlying mechanisms to manage and optimize etch rates in practical applications are also demonstrated by these subtle interplays between various chemical species [88,89].

Our own observations, as indicated in Figure A5b, showed an increase in etching rate at lower cryogenic temperatures. A comprehensive study of the experimental data indicates that with a decrease in temperature, etching shows better efficiency. For instance, at −110 °C, the etching speed significantly improved and reached an extraordinary level of 2.5 µm/min. This change in rates with temperature demonstrates how complex cryogenics is able to influence the dissolution process.

### Appendix B.6. Etching Profiles

The dynamic relationship between temperature and etching profile is illustrated in Figure A6, following a detailed investigation of the subtle effects of temperature variations on the etching rate. This figure provides a thorough understanding of the numerous nuances of the process by carefully comparing the etching profiles at different temperatures. A pattern becomes apparent upon closer examination.

The cryogenic temperature drops from 20 to −20, −70, and −120 °C, and over-etching angles rise progressively to 51°, 69°, 83°, and 87°, in that order. The relationship between temperature modulation and the resulting over-etching properties is clearly demonstrated by this astute finding. The etching profile has a notable positive taper, as revealed by the examination conducted at room temperature (20 °C) and at somewhat lower temperatures (−20 °C). The pronounced over-etching patterns and discernible increase in side wall roughness attest to this. These results indicate how the overall geometry and texture of the etched patterns are affected by even small changes in temperature throughout the etching process. On the other hand, a different pattern appears when the temperature drops even lower. The tendency toward vertical etching emerges at −120 °C, and the over-etching decreases. Together with this radical change in etching behavior, soft side walls occur, emphasizing the complex relationship between temperature and the structural properties of the etched patterns.

## Appendix C. Fresnel Zone Plate Parameters

As seen in Table A3, the diameter D and focal length f of a Fresnel lens, which characterize its optical performance, are defined by the radiation wavelength λ, the total number of zones N, and the outermost zone area Δr [32].

**Table A3:** Fresnel zone plate parameters.

Parameter	Notation	Values
Radiation wavelength	λ	1.03 nm (1.2 keV)
Outer zone width	$\Delta r_{n}=\frac{\lambda f}{2r_{n}}$	50 nm
Number of zones	*N*	100
Focal length	$f=\frac{r_{n}^{2}}{n\lambda }$	970.87 µm
Diameter	D=4NΔr	20 µm
Numerical Aperture (1st order)	NA = $\frac{\lambda }{2\Delta r}$	0.0103
Spatial Resolution (1st order)	$\delta_{\mathrm{res}}=1.22\Delta r$	61 nm
